# Natural Selection Shaped Codon Usage Patterns in Wheat Dwarf Virus in Triticale

**DOI:** 10.3390/biology14050524

**Published:** 2025-05-09

**Authors:** Jiuli Wang, Xinhang Lu, Jiaying Dong, Jiaqian Liu, Borui Guo, Chen Zhang, Jing Liu, Hongxia Wang

**Affiliations:** 1 College of Ecological Environment and Resources, Qinghai Minzu University, Xining 810007, China; wang_jiul@163.com (J.W.); xiaoxiao59408@163.com (X.L.);; 2 State Key Laboratory of Tibetan Medicine Research and Development, Qinghai University, Xining 810016, China

**Keywords:** codon usage, wheat dwarf virus, triticale, genome

## Abstract

Wheat dwarf virus (WDV) poses significant threats to gramineous crops, making it crucial to explore its codon usage patterns and evolutionary dynamics for effective disease control. This study analyzed ten WDV isolates, including two from triticale (WDVT_117 and WDVT_118), using metrics such as relative synonymous codon usage (RSCU), effective number of codons (ENC), codon adaptation index (CAI), and codon bias index (CBI). Neutrality plots, ENC-plots, and PR2-plots were employed to assess the role of mutation and selection. Results revealed weak codon preference in triticale-derived strains (CAI: 0.145–0.269; CBI: −0.042–0.111; ENC > 40), with hierarchical GC content. Neutrality analysis and ENC-plot distributions indicated natural selection as the dominant force, supported by T/C bias at the third codon position (PR2-plot). Shared optimal codons UUC and UAC in highly expressed genes may imply a potential significant role in virus adaptation. RSCU-based clustering and MP phylogenetic analysis revealed that WDVT strains form a distinct cluster with elevated genetic diversity, potentially driven by genomic recombination in the synthetic host. These findings demonstrate that WDVT balances mutational constraints and host adaptation through selective codon optimization. This study provides a foundation for codon-based antiviral research and the development of agricultural strategies to combat WDV infections.

## Simple Summary

In this study, we set out to explore the codon usage patterns and evolutionary dynamics of the wheat dwarf virus (WDV) in triticale, a crop combining wheat and rye. We analyzed ten WDV isolates, including two from triticale (WDVT), using various metrics such as relative synonymous codon usage (RSCU), effective number of codons (ENC), codon adaptation index (CAI), and codon bias index (CBI). Our results revealed weak codon preference in triticale-derived strains, with hierarchical GC content. Neutrality analysis and ENC-plot distributions indicated natural selection as the dominant force. We identified shared optimal codons UUC and UAC in highly expressed genes, which may play a significant role in virus adaptation. Furthermore, we found that WDVT strains form a distinct cluster with elevated genetic diversity, potentially driven by genomic recombination in the synthetic host. These findings provide a foundation for codon-based antiviral research and the development of agricultural strategies to combat WDV infections.

## 1. Introduction

Triticale (× *Triticosecale* Wittm. ex A. Camus) is a crop artificially combined from the genera of wheat (*Triticum* spp.) and rye (*Secale graine*) by intergeneric sexual hybridization and doubling of the chromosome number of the hybrids, which has been widely planted all over the world [1,2]. It combines the high yield and high quality of wheat and the disease resistance, strong resistance, and high lysine content of rye, with huge hybrid advantages, and is a high-quality forage crop after alfalfa and forage corn [3,4]. Currently, triticale mainly exists as hexaploid (AABBRR) and octoploid (AABBDDRR) types [5,6]. The hexaploid type is formed by crossing tetraploid wheat (AABB) with rye and then doubling the chromosomes of the F_1_ hybrid, resulting in 21 pairs of chromosomes, 7 of which come from rye [7]. The octoploid type is created by crossing hexaploid common wheat (AABBDD) with rye and then doubling the chromosomes of the F_1_ hybrid. Octoploid triticale retains the high yield and seed quality of wheat, as well as the strong stress tolerance and high lysine content of rye, and can adapt to different climates and environmental conditions [3,4,5,6].

Wheat dwarf virus (WDV), a member of the Geminiviridae family and *Mastrevirus* genus, is a pathogen of gramineous plants transmitted by the leafhopper (*Psammotettix alienus*) in a persistent, non-propagative manner [8]. It is a twinned icosahedral virus with a circular ssDNA genome that encodes four proteins: V1 (movement protein), V2 (coat protein), and the replication-associated proteins RepA and Rep [8,9]. First found in western Czechoslovakia in 1961 [10], WDV has since been confirmed in Africa, Europe, and Asia [9,11]. It mainly affects gramineous crops like wheat (*Triticum aestivum*), barley (*Hordeum vulgare*), oat (*Avena sativa*), and triticale, causing severe stunting, leaf yellowing, reduced tillering, and significant yield losses [8,11,12].

In the genetic code, 61 codons encode the standard 20 amino acids (with 3 additional codons for termination). Tryptophan and methionine are each encoded by a single codon, while the remaining 18 amino acids are typically encoded by multiple synonymous codons. The usage of synonymous codons is not random, leading to differences in usage frequency, known as synonymous codon usage bias (SCUB) [13,14,15]. SCUB may be caused by various factors during evolution and also serves as a fine-tuning mechanism for gene expression [14,16]. It is widespread in different species, tissues, and genes and is significant for gene expression regulation, genome evolution, and bioinformatics research [15,17,18,19]. For viruses, codon usage bias is closely related to natural selection, mutation pressure, optimal host selection, and drug sensitivity [20,21,22].

The SCUB of WDVT has not been reported to date. To address this gap, this study compared and analyzed the codon usage characteristics and driving factors of two available WDVT genomes, aiming to reveal WDVT’s codon usage bias and explore whether mutation or selection is the main driver of these bias.

## 2. Materials and Methods

### 2.1. Acquisition of Genetic Information of WDV in Triticale

This study retrieved the genomic sequences of all publicly available wheat dwarf virus isolates from triticale (WDVT) from the NCBI database (https://www.ncbi.nlm.nih.gov/, accessed on 13 November 2024), totaling two. Additionally, we obtained eight other isolates from diverse hosts and the intermediate vector, the leafhopper (*Psammotettix alienus*). These eight isolates encompass all currently known and publicly documented host types, including the intermediate host. Table 1 presents the details of these isolates (Table 1).

The GenBank Feature Extractor tool in the Sequence Manipulation Suite (SMS) (https://www.bioinformatics.org/sms2/genbank_feat.html; accessed on 13 November 2024) was used to extract protein coding sequences (coding sequence; CDS) from the wheat dwarf virus genome of wheat rye origin.

### 2.2. Codon Base Composition Analysis

CUSP software (https://www.bioinformatics.nl/cgi-bin/emboss/cusp, accessed on 14 November 2024) was used to calculate the GC frequencies at the three positions (GC1, GC2, and GC3) of each codon in the coding sequences (CDSs), the average GC frequency at the third position (GCall), and the frequency of GC at the third position of synonymous codons (GC3s). Additionally, the frequencies of each base (A3s, T3s, C3s, and G3s) at the third position of codons in the CDS were calculated using the Condon W1.4.2 software [23].

### 2.3. Calculation of Codon Usage Indexes

Relative Synonymous Codon Usage (RSCU) measures the usage frequency of a specific synonymous codon relative to its expected frequency [24]. The expected frequency is the average usage of all codons for the amino acid encoded by that codon. An RSCU of 1 indicates no usage preference, while values greater than 1 suggest higher usage frequency and values less than 1 indicate lower usage frequency.

The effective number of codons (ENC) reflects the degree of codon usage bias in a gene, ranging from 20 to 61 [25]. An ENC of 20 means only one codon is used for each amino acid, while an ENC of 61 indicates that all codons are used equally. Lower ENC values signify stronger codon usage bias.

The codon adaptation index (CAI) assesses the similarity between the codon usage of a gene and the optimal codon usage of the host organism, with values ranging from 0 to 1 [26]. A higher CAI value indicates better adaptation of the virus to the host’s codon usage.

The codon bias index (CBI) quantifies the extent to which a gene uses a set of optimal codons [27]. In genes with extreme codon bias, CBI equals 1.0, while in genes with random codon usage, CBI equals 0.0. Negative CBI values can occur due to random variations. CBI reflects the composition of highly expressed codons in a gene and correlates well with ENC values for the host’s own genes, indicating the potential expression level of foreign genes in the host.

The CDS sequences of the two WDVT were combined and analyzed using Condon W1.4.2 to calculate the overall RSCU and generate RSCU bar charts. The RSCU, ENC, CAI, and CBI values for each CDS of the two WDVT were also calculated using Condon W1.4.2. Finally, Pearson correlation analysis was performed to examine the correlations between CAI, CBI, ENC, GC1, GC2, GC3, and CDS length for the two strains.

### 2.4. Neutrality Plot Analysis

The neutrality plot is used to analyze the correlation between the average GC content at the first and second codon positions (GC12) and the GC content at the third codon position (GC3). This helps in understanding the factors affecting codon usage patterns and biases [28,29]. In this analysis, GC12 and GC3 values are plotted on the y-axis and x-axis, respectively, to create a two-dimensional scatter plot. The correlation between the two variables is analyzed by calculating the slope of the regression curve. A slope close to 1 indicates a significant correlation between GC12 and GC3, suggesting that codon usage is more influenced by mutation. Conversely, a slope close to 0 indicates no significant correlation, implying that natural selection is the primary factor affecting codon usage. Genes influenced primarily by mutation will be distributed along the diagonal, while those more affected by selection pressure will be scattered around the diagonal [29,30].

### 2.5. ENC-Plot Analysis

The ENC-plot analysis is a method used to assess the impact of base composition on codon usage bias [31]. This is achieved by creating a two-dimensional scatter plot with GC3 on the x-axis and ENC on the y-axis. It helps determine whether codon usage in genes is influenced by mutation or selection. If the genes are distributed along or fall near the standard curve (1), it indicates that they are affected by mutation only; otherwise, it indicates that they are affected by selection [32].

(1) $$ENC=2+{GC}_{3}+\frac{29}{{GC}_{3}^{2}+{\left(1-{GC}_{3}\right)}^{2}}$$

### 2.6. PR2-Plot Analysis

The PR2-plot is used to assess whether mutation pressure and natural selection influence nucleotide composition in DNA strands. In this plot, the y-axis represents the A3/(A3 + T3) ratio, and the x-axis represents the G3/(G3 + C3) ratio. Each point on the plot corresponds to a gene’s base composition. The center of the plot represents the state where codons are used without preference (A=T and C=G). The vector distance of other points from the center indicates the direction and extent of the bias [29].

### 2.7. Identification of the Optimal Codons

Optimal codons are most frequently used in highly expressed genes of a species [31]. Referencing the ENC, we select the top and bottom 10% of genes to create high RSCU datasets (hRSCU) and low RSCU datasets (lRSCU). The RSCU values are calculated using Codon W1.4.2 software, and the ΔRSCU (ΔRSCU = hRSCU − lRSCU) is then determined. Codons with ΔRSCU ≥ 0.08 in high- and low-expression gene sets, and with RSCU > 1, are identified as optimal codons [32].

### 2.8. RSCU Clustering and Phylogenetic Analysis

The RSCU values of each gene of 10 different sources of WDV strains were calculated separately, followed by the calculation of the distance between the data using the maximum parsimony (MP) difference method and the plotting of tree clusters and heat maps of RSCU values. The complete sequences of the WDV genomes of the 10 WDV strains were collated, and a phylogenetic tree (bootstrap = 1000) was constructed based on the MP method using MEGA11 [33].

## 3. Results

### 3.1. Codon Composition

**Figure 1:**
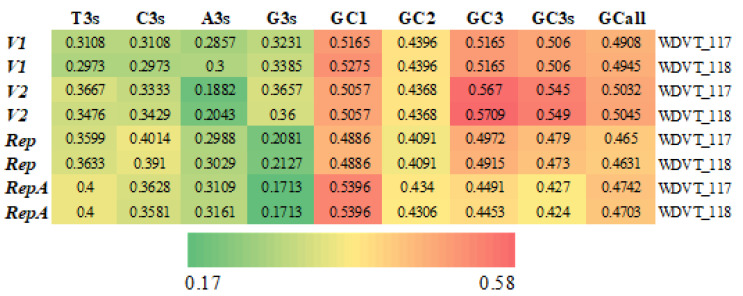
Frequency heat map of bases.

The two WDVT genomes, WDVT-117 and WDVT-118, have the same gene types and lengths (Table 2). Key indicators of their codon base composition, including A3s, T3s, C3s, G3s, GC1, GC2, GC3, GC3s, and GCall, were calculated (Figure 1). Notably, G3s and A3s are lower than T3s and C3s, and GC1 is generally higher than GC2 and GC3.

### 3.2. Synonymous Codon Usage Features

**Figure 2:**
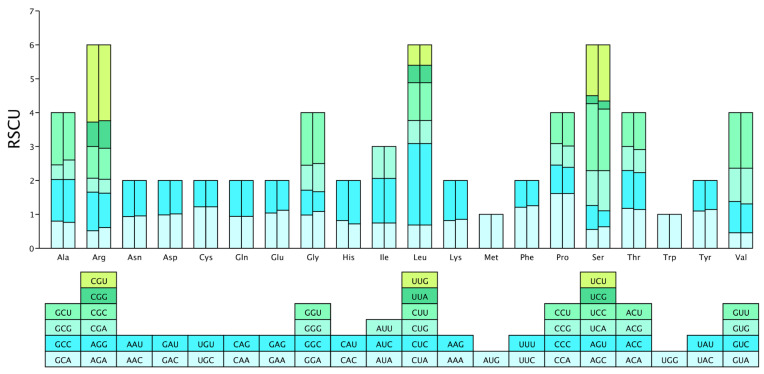
RSCU bar chart. Each amino acid corresponds to two stacked bars in the chart: the left bar represents WDVT_117, and the right bar represents WDVT_118, showing the RSCU values of synonymous codons for each amino acid in the respective strains. Each color in the stacked bars corresponds to a specific synonymous codon for the amino acids. The same colors are used in the RSCU bar chart above to represent the RSCU values of the respective codons.

On the genomes of the two WDVT strains, Arg, Leu, and Ser have the most synonymous codons, each encoded by six codons; Ala, Gly, Pro, Thr, and Val are each encoded by four codons; Ile is unique in being encoded by three codons; Met and Trp are each encoded by a single codon; and the remaining amino acids are each encoded by two codons (Figure 2). The RSCU values of the codons in the genomes of the two wheat dwarf virus strains show a high degree of similarity, as intuitively displayed by the RSCU bar chart, with some differences also present.

**Figure 3:**
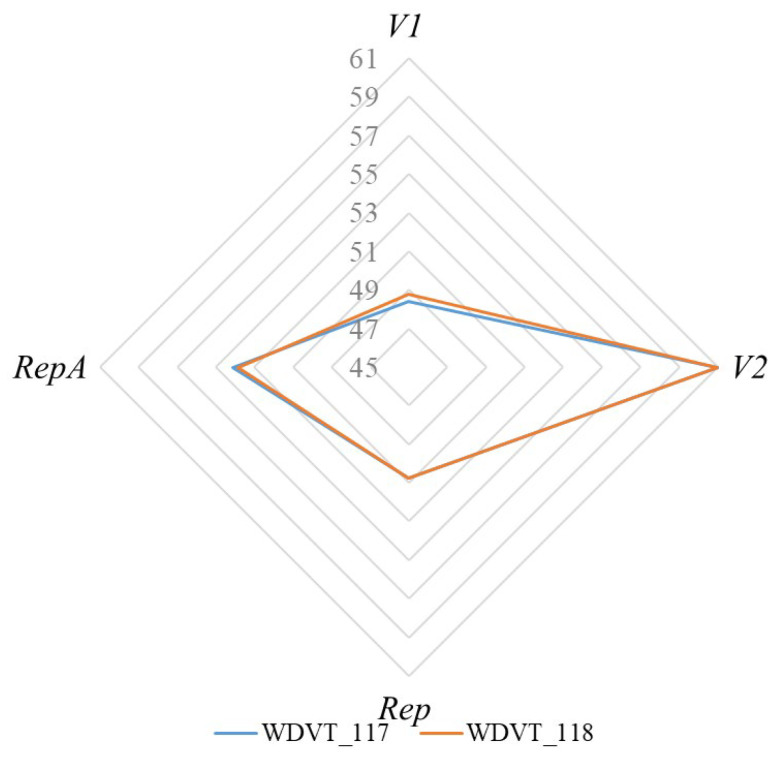
ENC radar map.

The effective number of codons (ENC) is used to assess codon usage bias. The ENC values for the CDSs of WDVT_117 and WDVT_118 ranged from 48.39 to 61 and 48.76 to 61, with average values of 53.58 and 53.60, respectively. The highest ENC value (61) was found in the *V2* gene for both strains, while the lowest values were in *V1* (48.39 for WDVT_117 and 48.76 for WDVT_118) (Figure 3).

In WDVT_117, the CAI of the four CDS ranges from 0.155 to 0.267 (average: 0.237), and the CBI ranges from −0.026 to 0.1 (average: 0.052). In WDVT_118, the CAI ranges from 0.145 to 0.269 (average: 0.236), and the CBI ranges from −0.042 to 0.111 (average: 0.056) (Table 3).

### 3.3. Amino Acid Usage

**Figure 4:**
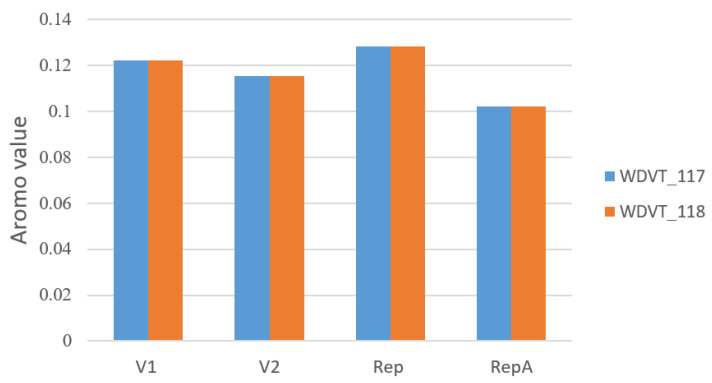
Bar chart of Aromo values.

Aromatic Amino Acid Proportion (Aromo): Aromo measures the proportion of aromatic amino acids (Phe, Tyr, Trp) in gene translation, indicating their relative content in proteins. In the four peptides V1, V2, Rep, and RepA, the aromatic amino acid proportions are similar between the two viral strains. WDVT_117 and WDVT_118 show similar Aromo features: RepA has the lowest Aromo, while Rep has the highest. Aromo values range from 0.102 to 0.129 (Figure 4).

**Figure 5:**
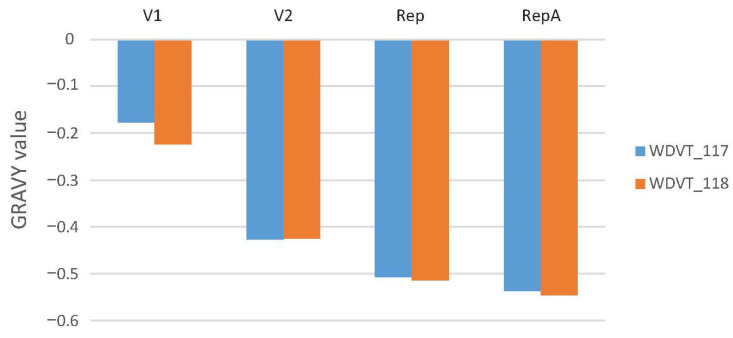
Bar chart of GRAVY values.

Grand Average of Hydropathicity (GRAVY): GRAVY measures protein hydrophilicity or hydrophobicity. Negative GRAVY values for all categories in both strains indicate overall hydrophilicity. In V1 protein, WDVT_118 has a lower value than WDVT_117, indicating slightly stronger hydrophilicity. In V2 protein, WDVT_117 has a lower GRAVY value than WDVT_118, suggesting more hydrophilic amino acids or fewer hydrophobic ones. In Rep, WDVT_118 has a lower value, indicating more hydrophilic amino acids. In RepA, WDVT_118 has a lower value, indicating stronger hydrophilicity. Data show that WDVT_118 is more hydrophilic than WDVT_117 (Figure 5).

### 3.4. Correlation Among Indexes

**Figure 6:**
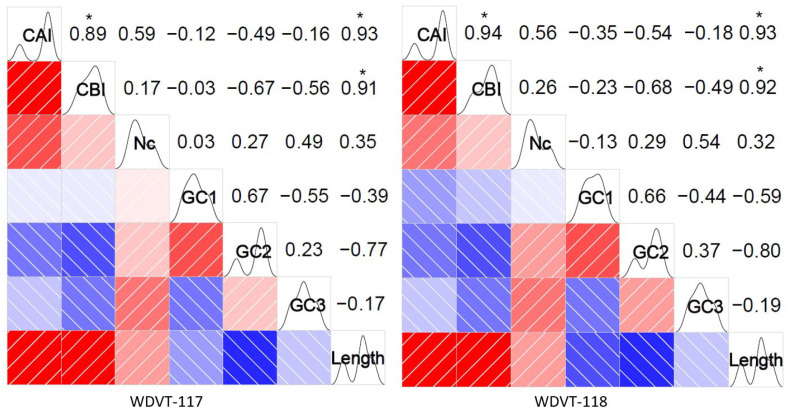
Correlation analysis of CAI, CBI, ENC, GC1, GC2, GC3, and length. * indicates significant correlation at the 0.05 level (*t*-tests). Red indicates a positive correlation, and blue indicates a negative correlation. The intensity of the color represents the strength of the correlation.

In the correlation matrix generated by Pearson’s method (Figure 6), red denotes a positive correlation and blue denotes a negative correlation. Values closer to 1 indicate stronger correlation, with 0.85–1.0 considered very strong. Analysis of WDVT-117 and WDVT-118 revealed a high degree of consistency in the correlation between codon-usage-related indexes in their genomes:

(a) CAI is very strongly positively correlated with CBI and length (significant at *p* < 0.05, *t*-tests), moderately positively correlated with ENC (significant at *p* < 0.05), and moderately negatively correlated with GC2. It has very weak negative correlations with GC1 and GC2.
(b) CBI is very weakly positively correlated with ENC, very weakly negatively correlated with GC1, strongly negatively correlated with GC2, moderately negatively correlated with GC3, and very strongly positively correlated with length (significant at *p* < 0.05).
(c) ENC has very weak positive and negative correlations with GC1, weak positive correlations with GC2 and length, and a moderate positive correlation with GC3.
(d) GC1 is strongly positively correlated with GC2, moderately negatively correlated with GC3, and weakly negatively correlated with length.
(e) GC2 has weak positive correlations with GC3 and length and strong negative correlations with length.
(f) GC3 has very weak negative correlations with length.

### 3.5. Neutrality Plot Analysis Reveals Natural Selection Dominance

**Figure 7:**
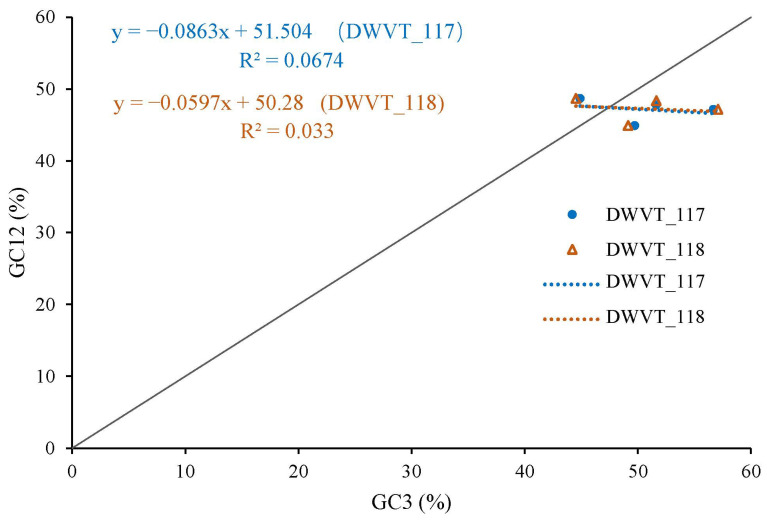
Neutrality plot analysis of WDV from triticale.

The magnitude of natural selection and mutation pressure in CUB was examined using a neutrality plot (GC12 vs. GC3). The slopes of the two regression lines (WDVT_117: y= −0.0863x + 51.504; WDVT_117: y = −0.0578x + 50.28) were close to zero, with R^2^ values of 0.0674 and 0.033, respectively (Figure 7). The narrow range of GC3 values and the absence of a significant correlation between GC12 and GC3 indicate the minimal influence of mutation pressure. This weak negative correlation suggests that natural selection predominantly shapes the codon usage pattern.

### 3.6. ENC-Plot Analysis Reveals Selection Pressure in WDV Codon Usage

**Figure 8:**
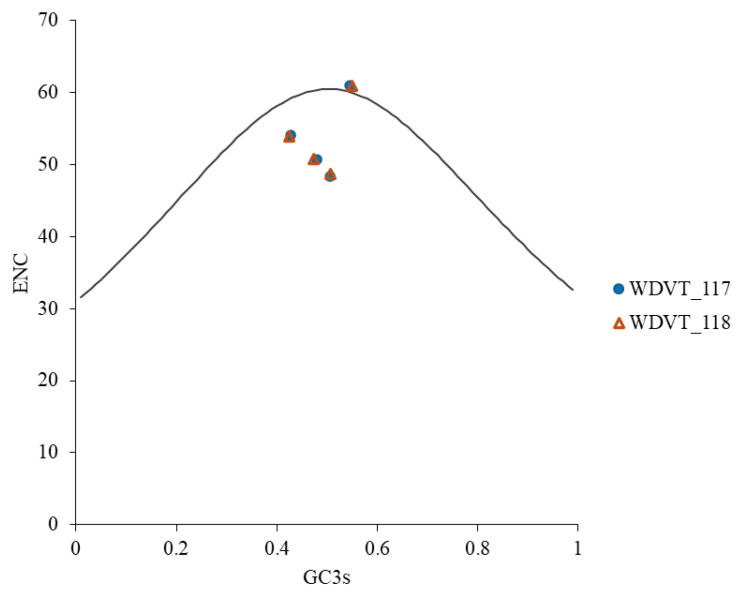
ENC-plot analysis of WDV proteins from triticale.

ENC-plot analysis, with GC3s on the x-axis and ENC on the y-axis, was used to evaluate the influence of natural selection and mutation on codon usage bias in WDV from triticale. Most genes were found below the standard curve (Figure 8), suggesting that codon usage bias is influenced by both mutation and natural selection, with the latter being more significant.

### 3.7. PR2-Plot Analysis Reveals Codon Usage Bias

**Figure 9:**
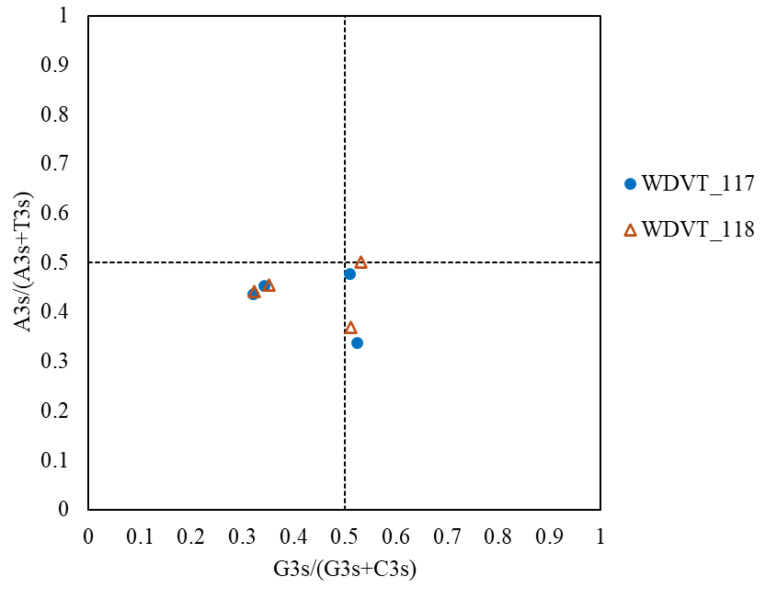
PR2-plot analysis of WDV proteins from triticale.

PR2-plot analysis further investigated codon usage bias in WDV proteins from triticale. Results showed that the four genes are mainly distributed with A3s/(A3s + T3s) < 0.5, with vectors biased downward and to the sides, more so to the left (Figure 9). This indicates a higher content of T and C in the third base of synonymous codons, revealing imbalanced usage frequencies of codon bases A3, T3, G3, and C3.

### 3.8. Optimal Codons

Screening by ENC identified *V2* as the low-expression gene and *V1* as the high-expression gene in both viral strains. RSCU values of *V2* and *V1* codons were used to establish low-expression (lRSCU) and high-expression (hRSCU) codon sets, respectively. ΔRSCU values were then calculated. The optimal codons of WDVT-117 and WDVT-118 are both UUC and UAC (Table 4).

### 3.9. Phylogenetic Analysis and RSCU Clustering

**Figure 10:**
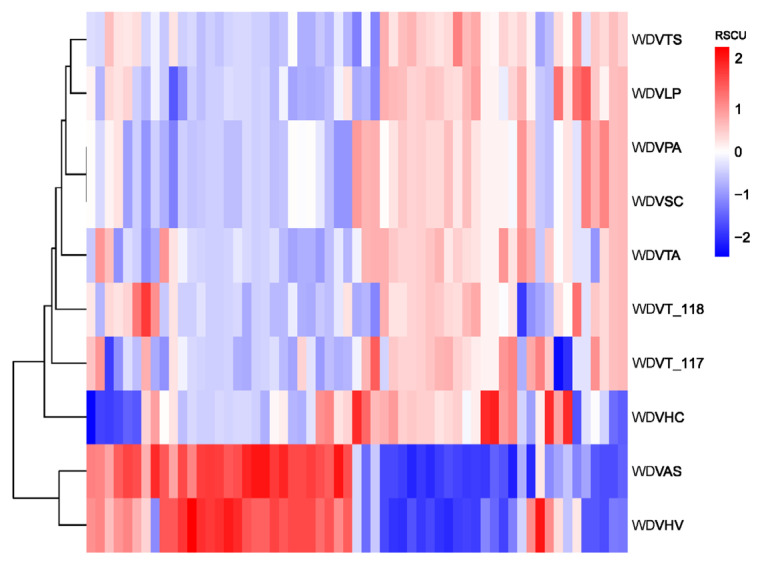
Clustering based on RSCU values.

**Figure 11:**
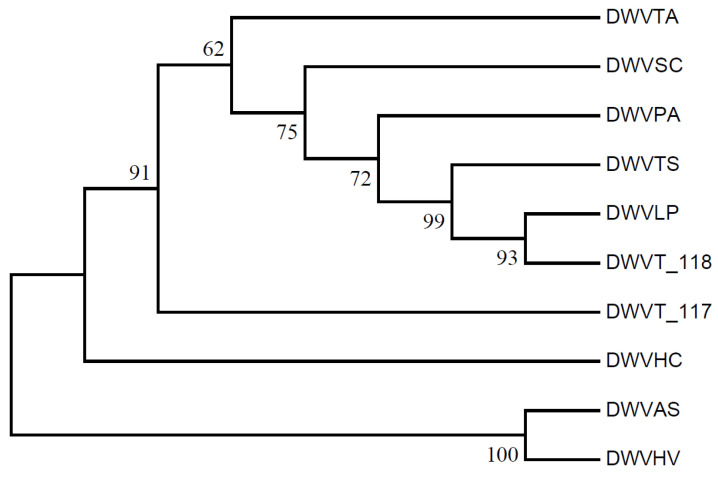
Phylogenetic tree of WDV strains.

Clustering based on RSCU values of genes from ten WDV strains revealed that the barley and oat isolates (WDVHV and WDVAS) formed one cluster, while the others, including triticale isolates WDVT_117 and WDVT_118, formed another, with the barley grass isolate (WDVHC) at its base. Notably, WDVT_117 and WDVT_118 did not cluster together in subordinate branches (Figure 10). A phylogenetic tree constructed using the maximum likelihood method based on the genome sequences of the ten stains also showed WDVHV and WDVAS in one cluster, WDVHC in another, and WDVT_117 and WDVT_118 in separate subordinate branches (Figure 11).

## 4. Discussion

Codon usage bias is widespread in various organisms and plays a significant role in gene expression regulation and species evolution [13,14,15,16,17,18,19,20,26,28,34,35]. According to the neutral evolution theory, the accumulation of high-frequency mutations in genomes may lead to the non-random distribution of specific codons [36,37,38,39,40]. Natural selection drives the formation of codon bias by optimizing translation efficiency, such as the adaptation of highly expressed genes to host tRNA abundance [34,40,41,42,43,44]. Additionally, genetic drift and interactions with multiple factors further shape codon usage patterns [14,15,16,45,46]. As obligate intracellular parasites, the codon usage evolution of viruses is closely related to host adaptation [47]. This study focuses on the codon usage characteristics of WDVT and systematically explores its evolutionary drivers.

The genomes of the 10 WDV strains analyzed range from 2734 to 2750 nt in length. Notably, the triticale-derived isolates WDVT_117 (2750 nt) and WDVT_118 (2748 nt) exhibit highly conserved gene compositions, with no significant differences in the GRAVY and Aromo index of their encoded proteins. Importantly, the G/A content at the third base position of their CDS is significantly lower than that of T/C (mean GC3s: 0.48), and follows a hierarchical decrease in the order GC1 > GC3 > GC2. This pattern is consistent with that observed in other viruses such as porcine circovirus [48], Zika virus [49], Pestivirus [50], and five species of silkworm viruses [51], suggesting similar evolutionary pressures or mechanisms in codon usage. However, it differs from viruses like rice yellow mottle virus [52]. Additionally, the relationship between GC1, GC2, and GC3 in different individuals of Soybean Mosaic Virus [53] shows marked differences, indicating that distinct viruses may exhibit significant variations in codon usage bias. This supports the hypothesis that the evolution of codon usage bias in plant DNA viruses is driven by host-selection pressure while retaining inherent genomic constraints [54].

The CAI values of WDVTs are generally low (WDVT_117: 0.155–0.267; WDVT_118: 0.145–0.269), indicating low matching with the codon usage of host high-expression genes. Research shows that *Mastrevirus* activates its gene transcription by hijacking the host’s epigenetic mechanisms, such as DNA methylation modification [55,56]. This mechanism may reduce the virus’s dependence on host optimal codons, instead balancing gene expression efficiency and host defense evasion through epigenetic regulation, leading to lower CAI values [56]. The *V1* gene has the lowest CAI values in both strains (WDVT-117: 0.155; WDVT-118: 0.145), much lower than other genes. This is likely related to the function of movement protein V1, which mediates virus particle transport between cells [26,57,58], so it does not need to match the codon usage of specific host high-expression genes. The CBI values of both strains are generally low (WDVT_117: −0.026 to 0.100; WDVT_118: −0.042 to 0.111), indicating poor adaptation to host-preferred codons [27]. ENC values are in the high range, above 40, with *V2* having the highest ENC value of 61 [25]. This aligns with the overall low CAI and CBI values, all suggesting weak codon usage bias. UUC and UAC were identified as the shared optimal codons in both WDVT strains, and its high usage in highly expressed genes implies a potential significant role in virus adaptation [47,48,49,50,51,52,53,54].

Despite the WDVT genome containing only four genes, the correlation coefficients among some codon indexes still reveal certain trends. The extremely strong positive correlations between CAI and CBI, and between CAI and CDS length, suggest that during gene elongation, viruses may need to co-optimize codon selection to maintain host tRNA compatibility [59]. The strong positive correlation between GC1 and GC2 indicates similar constraints on the first two bases, while the strong negative correlation between GC2 and CDS length suggests selective reduction in GC content during gene elongation. Results from neutrality plot, ENC-plot, and PR2-plot analyses consistently show that codon usage bias in WDVT is jointly influenced by mutation and natural selection, with natural selection being the dominant factor. Functional optimizations such as translation efficiency and tRNA abundance adaptation may drive the natural selection [24,25,59,60].

WDV has a broad spectrum of graminaceous crop infestation [61]. Cluster analysis based on RSCU values and the construction of an MP phylogenetic tree show that strains from barley and oats form one cluster, while those from triticale and others form another. This indicates that the genetic relationships among WDV strains do not align with the phylogenetic relationships of their hosts. This suggests that the WDV strains infecting triticale may have a high degree of genetic diversity, potentially accelerated by genomic recombination or epigenetic interactions in the artificially synthesized host triticale [11]. The biodiversity of the virus may be responsible for its broad attack capability.

Our current understanding of WDVT codon usage patterns remains constrained by two critical limitations: the scarcity of viral genomic data (only two triticale-derived isolates available) and the absence of a complete reference genome for its host, triticale. Viral codon usage is shaped by complex interactions between host-specific factors—including tRNA pool adaptation, transcriptional mechanisms, immune pressures, and genomic nucleotide composition—and evolutionary pressures from virus–host coevolution [62,63,64]. However, the lack of triticale’s genome impedes precise analysis of key indices such as the CAI and identification of optimal codons, which are essential for elucidating how WDVT balances mutational constraints with host adaptation strategies. For instance, comparative analysis of host and viral nucleotide composition could reveal whether WDVT codon bias aligns with triticale’s genomic signatures, a hallmark of host-driven selection. Therefore, future research must prioritize (1) expanding the WDVT genomic dataset to capture strain-specific variations and evolutionary trends, and (2) obtaining triticale’s genome to systematically evaluate how its genomic architecture and molecular machinery shape WDVT codon usage. These advancements will provide a robust framework to dissect the adaptive mechanisms driving WDVT evolution and its ecological success in triticale systems.

## 5. Conclusions

In this study, we investigated into the codon usage patterns and evolutionary forces at play in WDVT. Through comprehensive analysis of ten WDV isolates, including two WDVT, we uncovered several key insights. The WDVT strains exhibited weak SCUB, with natural selection emerging as the predominant force shaping their codon usage patterns, as evidenced by neutrality analysis and ENC-plot distributions. Shared optimal codons (UUC and UAC) in highly expressed genes were identified, potentially playing a crucial role in virus adaptation. Furthermore, WDVT strains formed a distinct cluster with elevated genetic diversity, possibly driven by genomic recombination in the synthetic host.

These findings enhance our understanding of the evolutionary dynamics of WDVT, providing a foundation for codon-based antiviral research. By clarifying the codon usage patterns and the factors influencing them, we can potentially develop more effective strategies to combat WDV infections in triticale. This is crucial given the importance of triticale as a high-quality forage crop with significant agricultural value. Our study also highlights the need for further research to expand the WDVT genomic dataset and obtain triticale’s genome to gain a more comprehensive understanding of the virus-host interactions and evolutionary mechanisms. Overall, this work contributes to the broader field of viral evolution and host-pathogen interactions, offering valuable insights for agricultural research and crop protection.

## Figures and Tables

**Table 1:** Information on wheat dwarf virus genomes.

Host	Accession Number	Genome Length	Designation
*Triticum aestivum*	HF968638.1	2750 nt	WDVTA
*Hordeum vulgare*	HG422315.1	2735 nt	WDVHV
*Lolium perenne*	HG422316.1	2750 nt	WDVLP
*Secale cereale*	HG422318.1	2749 nt	WDVSC
*Psammotettix alienus*	AM040733.1	2750 nt	WDVPA
*Avena sativa*	AM296018.1	2734 nt	WDVAS
*Triticum spelta*	KJ473696.1	2748 nt	WDVTS
*Hordeum vulgare* var. *coeleste*	KJ536149.1	2750 nt	WDVHC
× *Triticosecale*	KJ473702.1	2750 nt	WDVT_117
× *Triticosecale*	KJ473703.1	2748 nt	WDVT_118

**Table 2:** CDS of wheat dwarf virus from triticale.

Gene	Production	Length (nt)
*V1*	movement protein	273
*V2*	coat protein	783
*Rep*	replication-associated protein	1056
*RepA*	replication-associated protein A	795

**Table 3:** CAI and CBI for each gene of WDVT.

Gene	WDVT_117		WDVT_118	
	CAI	CBI	CAI	CBI
*V1*	0.155	−0.026	0.145	−0.042
*V2*	0.261	0.035	0.262	0.05
*Rep*	0.267	0.1	0.269	0.106
*RepA*	0.266	0.098	0.269	0.111
Average	0.237	0.052	0.236	0.056

**Table 4:** Analysis of optimal codons.

Amino Acid	Codon	WDVT_117			WDVT_118		
		hRSCU	lRSCU	ΔRSCU	hRSCU	lRSCU	ΔRSCU
Ala	GCA	0.5	0.8889	−0.3889	0.5	0.7059	−0.2059
Ala	GCC	2	0.8889	1.1111	2	0.9412	1.0588
Ala	GCG	0.5	0.6667	−0.1667	0.5	0.9412	−0.4412
Ala	GCU	1	1.5556	−0.5556	1	1.4118	−0.4118
Cys	UGC	0	1.2	−1.2	0	1.2	−1.2
Cys	UGU	2	0.8	1.2	2	0.8	1.2
Asp	GAC	1.5	0.875	0.625	1.5	0.875	0.625
Asp	GAU	0.5	1.125	−0.625	0.5	1.125	−0.625
Glu	GAA	0.6667	1.1	−0.4333	0.6667	1.2	−0.5333
Glu	GAG	1.3333	0.9	0.4333	1.3333	0.8	0.5333
Phe	UUC	1	1.0909	−0.0909	1	1.0909	−0.0909
Phe	UUU	1	0.9091	0.0909	1	0.9091	0.0909
Gly	GGA	0.5714	1.5	−0.9286	1.3333	1.5	−0.1667
Gly	GGC	0.5714	1	−0.4286	0	1	−1
Gly	GGG	0.5714	0.5	0.0714	0.6667	0.5	0.1667
Gly	GGU	2.2857	1	1.2857	2	1	1
His	CAC	0	0.75	−0.75	0	0.6667	−0.6667
His	CAU	0	1.25	−1.25	0	1.3333	−1.3333
Ile	AUA	1.8	0.75	1.05	1.8	0.75	1.05
Ile	AUC	0	1.5	−1.5	0	1.5	−1.5
Ile	AUU	1.2	0.75	0.45	1.2	0.75	0.45
Lys	AAA	0.5	1.3333	−0.8333	0.5	1.3333	−0.8333
Lys	AAG	1.5	0.6667	0.8333	1.5	0.6667	0.8333
Leu	CUA	0.6	0.5	0.1	0.6	0.5	0.1
Leu	CUC	0.6	3	−2.4	0.6	3	−2.4
Leu	CUG	0.6	0.25	0.35	1.2	0.25	0.95
Leu	CUU	1.2	1.5	−0.3	1.2	1.5	−0.3
Leu	UUA	1.8	0.25	1.55	1.8	0.25	1.55
Leu	UUG	1.2	0.5	0.7	0.6	0.5	0.1
Met	AUG	1	1	0	1	1	0
Asn	AAC	0	1	−1	0	1.1111	−1.1111
Asn	AAU	2	1	1	2	0.8889	1.1111
Pro	CCA	0	2	−2	0	2	−2
Pro	CCC	1.1429	0.6667	0.4762	1.1429	0.6667	0.4762
Pro	CCG	1.7143	0.2222	1.4921	1.7143	0.2222	1.4921
Pro	CCU	1.1429	1.1111	0.0318	1.1429	1.1111	0.0318
Gln	CAA	1.3333	1.2727	0.0606	1.3333	1.2727	0.0606
Gln	CAG	0.6667	0.7273	−0.0606	0.6667	0.7273	−0.0606
Arg	AGA	1	0.75	0.25	0.8571	0.75	0.1071
Arg	AGG	3	0.75	2.25	2.5714	0.75	1.8214
Arg	CGA	1	0	1	0.8571	0	0.8571
Arg	CGC	0	1.125	−1.125	0	1.125	−1.125
Arg	CGG	1	0.375	0.625	1.7143	0.375	1.3393
Arg	CGU	0	3	−3	0	3	−3
Ser	AGC	0	0.6207	−0.6207	0	0.6207	−0.6207
Ser	AGU	0	0.6207	−0.6207	0	0.4138	−0.4138
Ser	UCA	0	1.0345	−1.0345	0	1.2414	−1.2414
Ser	UCC	6	1.8621	4.1379	6	1.6552	4.3448
Ser	UCG	0	0	0	0	0	0
Ser	UCU	0	1.8621	−1.8621	0	2.069	−2.069
Thr	ACA	1.3333	1.4118	−0.0785	1.3333	1.3333	0
Thr	ACC	2	0.9412	1.0588	2	0.8889	1.1111
Thr	ACG	0	0.4706	−0.4706	0	0.4444	−0.4444
Thr	ACU	0.6667	1.1765	−0.5098	0.6667	1.3333	−0.6666
Val	GUA	0.8	0.3636	0.4364	0.8	0.3636	0.4364
Val	GUC	0	0.3636	−0.3636	0	0.3636	−0.3636
Val	GUG	1.6	0.7273	0.8727	1.6	0.7273	0.8727
Val	GUU	1.6	2.5455	−0.9455	1.6	2.5455	−0.9455
Trp	UGG	1	1	0	1	1	0
Tyr	UAC	1.6	1.1667	0.4333	1.6	1.1667	0.4333
Tyr	UAU	0.4	0.8333	−0.4333	0.4	0.8333	−0.4333

## Data Availability

The datasets used and/or analyzed during the current study can be obtained from the corresponding authors upon reasonable request. The datasets analyzed during the current study are available on the website at https://www.scidb.cn/en/s/Nr2eim (accessed on 12 December 2024).

## Abbreviations

The following abbreviations are used in this manuscript:

RSCU	Relative synonymous codon usage
WDV	Wheat dwarf virus
WDVT	Wheat dwarf virus isolates from triticale
CAI	Codon adaptation index
SCUB	Synonymous codon usage bias
CBI	Codon bias index
ENC	Effective number of codons
CDS	Coding sequences
Aromo	Aromatic amino acid proportion
GRAVY	Grand average of hydropathicity
MP	Maximum parsimony
